# Assessing the Current State of Lung Cancer Chemoprevention: A Comprehensive Overview

**DOI:** 10.3390/cancers12051265

**Published:** 2020-05-17

**Authors:** Md Ashraf-Uz-Zaman, Aditya Bhalerao, Constantinos M. Mikelis, Luca Cucullo, Nadezhda A. German

**Affiliations:** 1 Department of Pharmaceutical Sciences, Texas Tech University Health Sciences Center, Amarillo, TX 79106, USA; md.ashraf@ttuhsc.edu (M.A.-U.-Z.); Aditya.Bhalerao@ttuhsc.edu (A.B.); constantinos.mikelis@ttuhsc.edu (C.M.M.); Luca.Cucullo@ttuhsc.edu (L.C.); 2 Center for Blood-Brain Barrier Research, Texas Tech University Health Sciences Center, Amarillo, TX 79106, USA; 3 Center of Excellence for Translational Neuroscience and Therapeutics, Texas Tech University Health Sciences Center, Lubbock, TX 79430, USA

**Keywords:** oxidative stress, cigarette smoke, inflammation, tumorigenesis, repurposing, nicotine

## Abstract

Chemoprevention of lung cancer is thought to significantly reduce the risk of acquiring these conditions in the subpopulation of patients with underlying health issues, such as chronic obstructive pulmonary disorder and smoking-associated lung problems. Many strategies have been tested in the previous decades, with very few translating to successful clinical trials in specific subpopulations of patients. In this review, we analyze these strategies, as well as new approaches that have emerged throughout the last few years, including synthetic lethality concept and microbiome-induced regulation of lung carcinogenesis. Overall, the continuous effort in the area of lung chemoprevention is required to develop practical therapeutical approaches. Given the inconsistency of results obtained in clinical trials targeting lung cancer chemoprevention in various subgroups of patients that differ in the underlying health condition, race, and gender, we believe that individualized approaches will have more promise than generalized treatments.

## 1. Introduction

In 2020, the number of new cases of lung and bronchus cancer in the United States is predicted to be 228,820 cases, almost equally distributed between males and females [1]. Although this number is second to the estimated cases of breast cancer (279,100), the predicted death rate for lung cancer (59%) is well above any other cancer type [1]. The vast majority of patients with non-small cell lung cancer (NSCLC) and small cell lung carcinoma (SCLC) are smokers, current or former. A male smoker was shown to have 23 times more chance to acquire lung cancer when compared to never-smoking patients [2], all due to the accumulation of tobacco-induced genetic and epigenetic abnormalities in epithelial cells. Over the last several decades, the USA government launched an extensive and successful campaign to reduce the number of tobacco users among the general population. As part of tobacco smoking “optimization”, the recent trend of substituting the regular cigarettes for the electronic analogs (e-cigarettes) or vaping became popular under the assumption of reduced risk to lungs. After seven years of being on the US market, e-cigarettes became the most popular tobacco product amongst young population [3], with approximately 20% of high school students reporting its use [4]. However, the large number of vaping-induced acute lung damage cases prompted the Center for Disease Control and Prevention (CDC) to initiate an epidemiological investigation. As a result, respiratory distress caused by e-cigarettes was named as ***e****-cigarette, or **v**aping, product use–**a**ssociated **l**ung **i**njury* (EVALI). As of January 2020, 50 states reported 2711 confirmed cases of EVALI. Also, 60 deaths in 27 states and District of Columbia were due to this syndrome [5]. Many of the EVALI cases have pathologic features consistent with the ones present in chemical-induced pneumonitis. Although the prevalence of lung cancer in EVALI patients has not been reported yet, it is well recorded in patients with pneumonitis [6]. Hence, the e-cigarette–induced lung damage can be a significant risk factor for lung cancer development.

Tobacco use, although the most prevalent, is not the only cause of lung cancer. Among NSCLC patients, approximately 15% of men and 50% of women develop adenocarcinoma (ADC), a non-smoking-associated lung cancer [7]. Due to the difference in etiology, clinical symptoms, tumor biology, tumor microenvironment, sensitivity to chemotherapy, and treatment outcomes, non-smoking associated lung cancer is proposed to be a disease that is different from the smoking-induced lung cancer [8,9]. For example, non-smoking-associated lung cancer prevails in patients of Asian descent, mostly females [10,11]. As ADC affects patients of the younger age group, and it is sensitive to treatment with epidermal growth factor receptor (EGFR) -tyrosine kinase inhibitors, favorable outcomes are much more often than in the smoker subset of NSCLC cases [10]. Many developed countries report a significant decline in smoking rates. Thus, in these regions, we might see a prevalence of adenocarcinomas among new cases of lung cancers.

## 2. Lung Carcinogenesis

Lung cancer biology has been a subject of extensive studies for several decades. At the moment, it became evident that genetic and epigenetic pathways are very different between ADC and smoking-associated lung cancer [8]. Moreover, non-smokers and never-smokers develop lung cancer from the cells in the peripheral compartment of bronchioli and alveoli. In contrast, SCLC, squamous cell carcinomas (SCC), and approximately 20% of ADC develop in the central compartments of the bronchiole [12]. This difference may play a major role in identifying optimal chemoprevention pathways.

**Figure 1:**
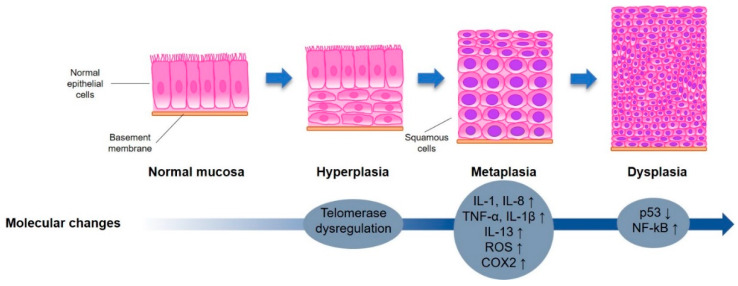
Stages of morphological cellular adaptations and molecular changes leading to lung cancer. Representative illustration highlighting morphological alterations of the epithelial cells during the gradual transition towards lung cancer and key molecular alterations contributing to this process.

Lung tumorigenesis in smokers is shown to be not only a multistep (Figure 1) but also a multicentric process, where tumors can develop simultaneously in multiple sites of the respiratory system [12]. The multistep process consists of a transition of a normal epithelial cell to the malignant state via stages of hyperplasia, metaplasia, and dysplasia [13,14,15]. The moderate-to-severe dysplasia is considered to be a pre-cancer state and is characterized by the presence of intraepithelial neoplasia (IEN)—a “non-invasive lesion with the genetic abnormalities, loss of cellular control functions and with phenotypic characteristics of invasive cancer” [15]. The World Health Organization defines three types of IENs in the lungs: squamous dysplasia and carcinoma in situ (CIS), atypical adenomatous hyperplasia (AAH), and diffuse idiopathic pulmonary neuroendocrine neoplasia [15]. As IEN is a good predictor of developing invasive cancer, its prevention and regression are hallmarks of chemoprevention clinical trials. In the subpopulation of smokers, the formation of IENs and initiation of lung cancer is often triggered by nicotine and tobacco-induced changes.

### 2.1. Role of Nicotine in the Onset of Lung Cancer

Nicotine-associated tumor progression occurs via nicotinic acetylcholine receptor (nAChR)-induced pathways. nAChRs are well present in the lung epithelial cells [16], with some (cm-nAChRs) being expressed on the cell membrane [17], and some (mt-)nAChRs) located on the mitochondrial outer membrane [18]. Natural ligand of these receptors, acetylcholine (ACh), is known to regulate a variety of cell signaling pathways responsible for cell apoptosis, differentiation, adhesion, and motility [19]. Due to higher receptor-binding affinity, nicotine replaces the acetylcholine and promotes the upregulation of genes responsible for lung cancer via increased interaction between (cm-)nAChRs and selected growth factors (EGF, VEGF, FGF, and IFG-I) [20]. By binding to (mt-)nAChRs, nicotine inhibits an initial step of apoptosis [21] and upregulates levels of ⍺7 mt-nAChR subtype, responsible for amplification of cancer-promoting signaling and selection for chemoresistant cells [17].

### 2.2. Role of Smoking-Associated Toxins in the Initiation of Lung Cancer

**Figure 2:**
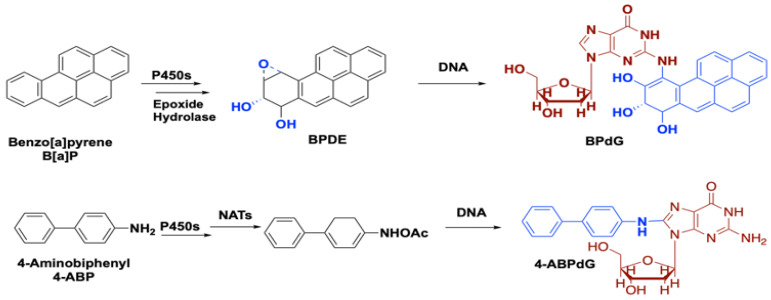
Formation of the DNA adducts by benzo[a]pyrene (B[a]P) and 4-aminobiphenyl (4-ABP).

**Figure 3:**
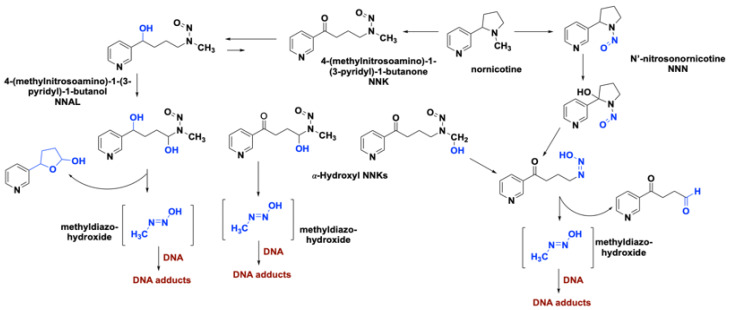
Role of nicotine-derived metabolites in the formation of DNA adducts.

Cigarette smoke contains about 8000 chemicals [22] with more than 60 having a human inhalation risk value for cancer [23]. Among these carcinogens, polycyclic aromatic hydrocarbons, aza-arenes, and *N*’-nitrosamines play a major role in lung carcinogenesis. Within the class of polycyclic aromatic hydrocarbons, benzo[a]pyrene (B[a]P) and 4-aminobiphenyl (4-ABP) are highly implicated in lung tumor formation via direct binding to DNA (Figure 2). Metabolic transformations of other nicotine-derived carcinogens, nitrosamine ketone (NNK), N-nitrosonicotine (NNN), and nitrosamine alcohol (NNAL), produce methyl diazohydroxide as a byproduct that forms various DNA adducts (Figure 3) [24,25]. It has been well documented that the level of DNA adducts in different tissues of smokers is much higher than in the ones obtained from non- or never-smokers, causing irreparable damage to the genetic material [15]. Not only cancerous cells but also histologically normal bronchial mucosa of smokers and formal smokers accumulate a large number of genetic and epigenetic changes. This phenomenon explains why former smokers remain in the high-risk group [26].

Although electronic cigarettes were developed to eliminate the risk associated with toxic tobacco combustion products, they still present a serious health threat. In addition to nicotine-derived carcinogens (NNK, NNN, NNAL), e-cigarette aerosol has been shown to contain ultrafine particles, benzene, formaldehyde, acetaldehyde, toluene, cadmium, propylene oxide, diethylene glycol, diacetyl and acetyl propionyl, carbonyls and any combination of flavoring chemicals [27], many of which cause EVALI symptoms. In addition, e-cigarettes contain and deliver considerably higher concentrations of nicotine in comparison to conventional cigarettes, increasing tumorigenesis via nAChR-induced signaling, as described above.

### 2.3. Bio-Molecular Pathways Involved in Lung Carcinogenesis

#### 2.3.1. Inflammation

There is a strong correlation between cigarette smoke-induced chronic inflammation of airways and lung carcinogenesis. [28] In part, it is supported by clinical studies on the effect of aspirin use on lung cancer incidents among regular smokers [29,30]. Repeated exposure to cigarette smoke triggers a pro-inflammatory response in lung epithelial cells [31] that releases chemostatic and toxic mediators, such as reactive oxygen species (ROS), pro-inflammatory cytokines (IL-1, IL-8, TNF⍺, IL-1β) and many other pathogenic factors. The presence of such intermediates causes the damage of the lung tissue by many different mechanisms, promoting malignancy. [29,32] Chronic inflammation also results in suppression of p53, a transcriptional regulator that maintains cellular homeostasis in response to various stress and controls. The loss of p53 function is explained by the accumulation of epigenetic and genetic mutations due to the persistence of DNA adduct formation [33]. In addition, inflammatory responses activate pathways regulated by the transcription factor nuclear factor-κB (NF- κB), causing further accumulation of DNA damage in p53 mutant cells [34]. These events result in the reprogramming of lung epithelial cells and the progression of tumorigenesis [35]. Another marker of carcinogenesis is cyclooxygenase-2 (COX-2); an inducible form of cyclooxygenases that gets upregulated in the lung by chronic inflammation [33]. This marker can be found in pre-cancerous and malignant cells, suggesting importance of COX-2-induced signaling for cancer initiation and progression.

#### 2.3.2. Oxidative Stress

Smoking alters levels of ROS in the lung epithelial cells via induction of chronic inflammation and by the direct presence of ROS in cigarette smoke. In a normal state, ROS act as signaling molecules that regulate several pathways, including the Wnt/β-catenin signaling pathway, jun N-terminal kinases, p38 mitogen-activated protein kinases (p38 MAPK) and others [36]. Overproduction of ROS in the presence of environmental toxins, smoke-related toxins, ionizing radiation, inflammatory cytokines, and growth factors leads to ROS-induced DNA damage, replication errors and genetic instability [36]. Further, under oxidative stress conditions, the guanine base (G) in genomic DNA forms 8-oxi-7,8-dihydroguanine (8-oxoG) [37]. The increased reactivity of G towards oxygen is due to its low oxidative potential. Formation of the 8-oxoG results in the G-C to T-A (G-T) transversion, mostly at the DNA regions enriched with the cytosine-guanidine sequences (CpG sites). This event is characteristic of oxidative DNA lesions and is known to be mutagenic [38]. The repair mechanism includes 8-oxoguanine DNA glycosylase (OGG1)-initiated base excision repair, where OGG1 is 8-oxoguanine DNA glycosylase. Interestingly, no differences in the OGG activity were detected between male/female and smokers/non-smokers [39]. However, clinical studies identified a correlation between the low activity of OGG and the risk of NSCLC [40], which was further amplified by the active smoking status of patients [41].

Lipid peroxidation is another event associated with oxidative stress and cancer initiation. During this process, cell lipids in the presence of ROS form two major end products, trans-4-hydroxy-2-nonenal (HNE) and malondialdehyde (MDA) [42]. Both HNE and MDA are implicated in carcinogenesis via the formation of DNA adducts.

#### 2.3.3. Proliferative/Growth Signaling

Nicotine is the primary addictive component in tobacco. Although nicotine by itself is not a carcinogen, it promotes tumor progression, metastasis, angiogenesis, and activation of anti-apoptotic pathways [43,44]. Nicotine, a high-affinity ligand for the nicotinic acetylcholine receptors (nAChRs), as well as its metabolites (NNAL, NNN, and NNK), were shown to regulate tumor progression in lungs via the nicotinic system [45,46,47] as discussed above. In addition to nAChR-modulated signaling, NNK has been reported to augment lung cancer cell proliferation through the Akt pathway [48].

EGFR signaling pathway with its two main branches, Ras/MAPK, and PI3K/Akt pathways, is one of the major proliferation pathways affected by lung carcinogenesis. Hirsch et al. reported EGFR overexpression in squamous cell and ADC subtype of NSCLCs at the level of 62% [49], whereas the National Comprehensive Cancer Network stated that over 80% of NSCLC patients have detectable levels of EGFR in tumors. Thus, EGFR mutations are one of the driving forces of lung tumorigenesis, making lung cancer cells dependent on the EGFR signaling for growth and survival [50]. This effect is known as “oncogene addiction” and provides bases for tumor-selective chemotherapy [51]. In the case of lung cancer, the mutations are often found in the kinase domain [52], and inhibition of EGFR signaling by means of monoclonal antibodies and small molecule reversible TK inhibitors (TKIs) become a popular approach [12] that was overshadowed by a fast development of resistance. Extensive work has shown the dependence of the observed outcomes for TKI-based treatment on many parameters, including number and type of mutations in EGFR, KRAS mutations, *MET* (the hepatocyte growth factor receptor) amplification, and others [53,54,55], suggesting a great need for individualized strategies in the treatment of lung cancer patients.

Lung pathogenesis is also associated with abnormalities in several other signaling pathways, each associated with a specific type of lung cancer. For example, mutations in the PI3K pathways are more characteristic for SCCs than ADCs, or SCLCs [12]. Anaplastic lymphoma kinase (ALK) fusion proteins are usually found in never-smokers, and lung carcinogenesis associated with the ALK-associated RAS signaling pathway [56] is often specific for the non-smokers with the ADC histology. Finally, abnormalities in KRAS signaling are associated with the carcinogens in smokers [12]. Interestingly, in lung tumors KRAS and EGFR mutations are mutually exclusive, as well as Ras and BRAF mutations [12].

#### 2.3.4. Downregulation of Protective Mechanism

p53 pathway is one of the major pathways downregulated during carcinogenesis. Its usual role is to prevent the accumulation of stress signals (DNA damage, hypoxia, oncogene activations), preventing a build-up of genetic instability and abnormalities in cells [33]. It has been shown that in smokers, the mutation of p53 is strongly associated with high level of benzo[a]pyrene-DNA adducts that induce mutations at guanine in codons 157, 248, 273 and 157.

Oxidative stress in cells caused by toxic exposure is handled by nuclear factor E2-related factor 2 (Nrf2) pathway that generates an antioxidant response [57]. The role of Nrf2 in lung carcinogenesis was demonstrated in vivo, where the presence of DNA adducts formed by benzo[a]pyrene, cigarette smoke and other toxins had a higher impact on the Nrf2-null mice than on the control group [45,58,59]. As discussed in the review by Lau A. et al. [60], Nrf2 controls expression of genes that can be grouped into three categories: intracellular redox-balancing proteins, phase II detoxifying enzymes, and transporters. To turn the expression of these genes on, Nrf2 translocates into the nucleus, where it binds to an antioxidant response element (ARE) sequence [61]. This process is controlled by Kelch-like ECH-associated protein 1 (Keap-1), a molecular switch that senses redox disbalance and translates the message to the cell via the presence of three key residues. Cysteine-151 was shown to be required for activating the Nrf2 pathway, whereas cysteine-273 and cysteine-288 are essential for repressing this signaling system off [62,63,64]. After the restoration of the redox balance, Keap1 promotes dissociation of Nrf2 from ARE, promoting its polyubiquitination and proteasomal degradation. In lung cancer patients, approximately 30% of NSCLCs are characterized by mutations in the Nrf2 signaling pathway, including Keap1 dysfunction. Keap1 dysfunction results to inhibition of Nrf2 polyubiquitination and degradation, its accumulation in the cytoplasm and finally to increased transcription of ARE-responsive genes [65]. Moreover, Keap1 loss was shown to promote cell migration and metastasis in vitro [66] and in vivo [67]. This pro-cancerous effect can be associated with the Nrf2 modulation of iron signaling [68]. It has been shown that by interfering with HMOX-1 and ferritin signaling, Nrf2 facilitates cancer proliferation and induces drug resistance. Since the presence of Nrf2 is essential for minimizing the toxic effect caused by the presence of carcinogens, while constitutively overexpressed Nrf2 promotes carcinogenesis and drug resistance, the maintenance of the careful balance in the NRf2 signaling can be one of the strategies for chemoprevention and cancer treatment.

## 3. Chemoprevention Strategies for Lung Cancer

Prophylactic use of apparently non-toxic substances, including natural, synthetic of biological agents, for cancer prevention, was termed chemoprevention [15]. There are many potential targets for chemoprevention agents, prophylaxis includes reversal, suppression, prevention, and delay of the initial phase of carcinogenesis or the progression of premalignant cells to cancer [69]. In the vast majority of reported cases, the focus is made on the modulation of known tumorigenic pathways, including EGFR signaling pathways, p53 cascade, inflammation, and oxidative stress. Currently, nature-derived compounds, dietary supplements, and synthetic molecules, either known chemotherapeutic agents or repurposed drugs, are major sources of chemopreventive compounds [15,70]. A growing understanding of the interplay between microbiome and process of host carcinogenesis provides novel opportunities for cancer interception [71]. The most studied tactics in this approach include the use of prebiotics, probiotics, and dietary changes to restore homeostasis and reverse/suppress the development of cancer [72,73]. Microbiome changes are also viewed as a potential biomarker in cancer development, suggesting that monitoring of these changes can be used for early detection of cancer [74,75]. Finally, one of the most recent strategies focuses on the selective elimination of premalignant cells by inducing apoptosis only in altered cells [76]. This synthetic lethality approach [77] offers the advantage of minimal toxicity to normal cells and, unlike all other chemopreventive strategies, can be delivered noncontinuously, reducing cost of treatment.

Lung carcinogenesis is a very lengthy process, with the estimated span of 20–30 years. The length of this process provides plenty of time for possible interception. Current research targets the possibility of preventing primary, secondary, and tertiary tumors in the lungs, with each of these areas having specific biomarkers [15,78]. For chemoprevention of primary lung cancer, the goal is to prevent the formation of the severely dysplastic lesions. An example of these lesions associated with lung cancer is the AAH [79]. It contains multiple genetic alterations characteristic of invasive adenocarcinoma (mutations in Kras, p53, EGFR, multiple chromosomal sites with loss of heterozygosity, and hypermethylation), and often is associated with the high-risk population, such as smokers [80,81]. Hence, AAH was proposed to be a biomarker for lung cancer initiation. However, the characterization of these lesions requires the use of bronchial biopsies that can altogether remove the premalignant lesion at baseline bronchoscopy. In this case, healthy tissue will replace the injury, altering the actual picture of lung carcinogenesis [82]; hence, this sampling technique raises some concerns. One of the alternative approaches for monitoring carcinogenic processes is to measure an expression of the proliferative marker Ki-67 in bronchial biopsies, which was shown to be upregulated in samples that lack premalignant histological changes and serves as an endpoint biomarker. Ki-67 is associated with decreased survival in NSCLC patients and can be identified by immunohistochemistry [83]. The goal of the secondary chemoprevention strategy is to suppress cancer development in a patient’s lung that has developed precursor lesions. Finally, tertiary cancer prevention aims at reducing the rate of recurring cancer.

To accommodate continuous treatment over the long period of time of the otherwise healthy population, chemo-preventive agents have to be effective and non-toxic, presenting a bottleneck in drug development. At the moment, several classes of agents with the potential to prevent cancer formation in a high-risk population were dismissed due to the observed side effects. For example, tamoxifen use in the prevention of breast cancer was greatly limited due to the presence of thromboembolic events and risks of uterine cancer [84], whereas the use of non-steroidal anti-inflammatory drugs (NSAIDs) is questioned due to the risk of gastrointestinal bleeding and hemorrhagic stroke [85]. Some strategies were proposed to overcome this issue. Luai Al Rabadi et al. [86], propose to use local delivery of agents, such as inhalation therapy for lung chemoprevention, to minimize potential side effects. In another comprehensive review [69], Xiangwei et al. have summarized research on using short-term intermittent approaches for cancer prevention, highlighting their potential in reducing the toxicity of many agents.

### 3.1. Modulation of Tumorigenic Pathways as a Chemoprevention Strategy in Lung Cancer

Broadly, this category of agents used for chemoprevention of lung cancer can be divided into three groups. The first group includes antioxidants to reduce the oxidative stress in lung tissue, such as vitamins, extracts of medicinal plants, and synthetic agents. The second group consists of compounds with anti-inflammatory properties, like NSAIDs and peroxisome proliferator-activated receptor (PPAR)-gamma agonists. Finally, inhibition of insulin-like growth factor (IGF) signaling via PI3K/Akt pathways [87], and inhibition of mTOR signaling [88] were associated with antiproliferative and apoptotic chemopreventive activities.

*Vitamins and minerals as chemopreventive agents*: The earlier studies on the chemoprotective effect of vitamins and minerals in the lungs were designed using data from similar trials targeting other organs. In 1986, the Cancer Institute of the Chinese Academy of Medical Sciences and the National Cancer Institute (NCI) conducted two large randomized, double-blind nutrition intervention trials in China: the General Population Trial (over 30,000 participants, vitamin combinations included retinol and zinc; riboflavin and niacin; vitamin C and molybdenum; and β-carotene, selenium, and vitamin E) and the Dysplasia Trial (3,318 participants, daily supplementation of 14 vitamins and 12 minerals versus placebo) [89]. The main objective was to understand the effect of daily consumption of vitamins and minerals on cancer-associated mortality, specifically esophageal and gastric cancer and results were positive for both types of cancers. Understanding that lung carcinogenesis process in smokers is partially attributed to tobacco-related oxidative stress, it was hypothesized that β-carotene, vitamin E, and selenium will also show chemopreventive effect in a population with high risk for lung cancer. Three large primary prevention studies were conducted to investigate this hypothesis [90]. The Alpha-Tocopherol Beta Carotene (ATBC) Study was conducted in Finland in 1994, where 29,133 male smokers (smoked ≥ 5 cigarettes/day) were given a dietary supplement containing either ⍺-tocopherol (50 mg/day), β-carotene (20 mg/day), both ⍺-tocopherol and β-carotene, or placebo for five to eight years [91]. The ⍺-tocopherol arm of the study showed no effect, but the use of β-carotene as a single agent or in combination with ⍺-tocopherol was linked with 17% increase in the lung cancer rate and 8% increase in the overall death rate. Furthermore, the ⍺-tocopherol group reported a higher incidence of hemorrhagic stroke [91]. In the follow-up study conducted after four years, the risk of lung cancer remained high among smokers of the β-carotene group and reduced for those who quit smoking within a four-year period [92].

The second trial, the β-Carotene and Retinol efficacy Study (CARET), enrolled 18,314 current and former smokers (male and female), as well as workers (male) with history of asbestos exposure. The randomized groups were given β-carotene (30 mg/day) and retinol palmate (25,000 IU) versus placebo. The study was stopped ahead of schedule (lasted from 1985 to 1996, stopped 21 months earlier than planned) due to increased incidence of death, lung cancer, and higher rate of cardiovascular disease-related mortality when compared to the placebo group [93]. A general population clinical trial, the Physician’s Health Study, was conducted in the USA in 1982 and involved 22,071 male physicians that were current smokers (11%) or former smokers (39%) at the beginning of the study. The regimen consisted of β-carotene (50 mg on alternate days) and a placebo. After 12 years, no effect was observed on any of the outcomes (malignant neoplasia, cardiovascular diseases, and death from all causes) [94,95]. Several reasons, such as lower than needed levels of β-carotene and retinol in lungs [96] and reduced ability of smokers to absorb ⍺-tocopherol [97], were proposed to explain why previous data with the β-carotene and ⍺-tocopherol did not duplicate in clinical trials for prevention of lung cancer. Overall, these studies highlight the need for having data from animal models and mechanistic studies prior to conducting clinical trials [90,94]. A poor translation of epidemiologic observation based on the complex diet to the clinical trials assessing the effect of a single compound as a chemopreventive agent can be seen as a significant limitation [98]. Obtained results also emphasized the uniqueness of smoke-induced carcinogenesis processes, which is further confirmed by the lack of any working chemopreventive strategies for lung cancer in smokers despite successful chemopreventive trials for other types of cancers [94].

Selenium (Se) is known to be involved in metabolic and oxidative processes. As a structural component of glutathione peroxidase, this element reduces oxidative stress, exhibiting cytoprotective properties. Some animal studies, as well as selected clinical trials, established the anticarcinogenic activity of this element [99]. However, a meta-analysis of 15 human studies, 29 reports, and 41 preclinical studies performed by Fritz, H. et al. [100], identified a correlation between the chemoprevention properties of this element and the lower baseline selenium level in participants (serum< 106 ng/mL). At the same time, selenium supplement did not modulate risk of lung cancer in current or former smokers with the normal baseline of selenium in serum [101]. Finally, selenium was investigated for the ability to prevent secondary tumors in patients with resected stage I non-small cell lung cancer [102]. In this double-blind placebo-controlled study (1772 patients), the selenium arm received a daily dose of selenized yeast (200 ug) for 48 months. Although no toxicity associated with the dose regimen was found, this phase 3 trial has shown no chemopreventive benefits in selenium supplementation when compared to the placebo arm of the study.

*Phytochemicals as chemopreventive agents:* Chemopreventive effect of phytochemicals is well known and these natural compounds can affect multiple cancer-related pathways. Interestingly, these effects can be either selective for cancer cells only [103], or/and observed at the non-toxic levels of compounds, significantly below their corresponding IC_50_ values [104]. Selected examples of phytochemicals and pathways they affect can be seen in the Table 1, whereas more comprehensive list of these agents and their activities in various in vitro and in vivo models can be found elsewhere [103,105,106,107,108,109]. Several in vivo models of lung cancer in A/J mice, induced via B[a]P, NDEA or NNK treatment conformed anti-carcinogenic potential of tea tree extract, ginseng, honiokial and chinese drug Zeng Sheng Ping that contains a mixture of six plants [107]. Currently, the U.S. National Library of Medicine contains two records on clinical trials that investigate the role of green (NCT00363805) and/or black teas (NCT02719860) in chemoprevention of lung cancer. Both of the studies are marked as completed; however, no literature reports containing the corresponding data and their analysis can be found. Unlike other types of the cancer, there is no clinical evidence supporting the role of phytochemicals in the prevention of lung cancer in the high-risk population [108]. However, there is ongoing recruitment for two clinical studies analyzing the chemopreventive potential of cruciferous vegetables in subjects at the higher risk of lung cancer. One of these clinical trials (NCT02999399) investigates the ability of glucobrassicin, a chemical found in Brussel sprouts, to modify the metabolism of the smoke-related polycyclic hydrocarbons (Figure 2) [110]. Another study (NCT03232138) is designed to monitor the effect of dietary sulforaphane [111] intake for a period of 12 months on the carcinogenesis process in the lungs of former smokers.

Finally, we would like to discuss a link between the use of curcumin and lung cancer development. Curcumin was shown to have anticancer and chemopreventive potential in different types of cancers, as discussed in several reviews [112,113,114]. Its relative non-toxicity allowed to overcome any biovailability concers, resulting in over 200 clinical trials investigating the protective role of curcumin against cardiovascular, metabolic, neurological, inflammatory and many other diseases [115]. It affects a wide range of cancer-related pathways, including inflammation, oxidative stress, apoptosis, and others. Due to its anti-inflammatory potential, curcumin was included in the clinical trial to investigate its benefits in the NSCLC patients undergoing treatment with the EGFR-TKIs (NCT02321293). At the moment of submission of this review, the National Cancer Institute has no reports on clinical trial analyzing the role of curcumin in the lung chemoprevention. Moreover, the Dance-Barnes et al. [116] have published results showing a significant increase in the number of lung lesions (adenomas and adenocarcinomas) and progression to the later stage of lesions in animals treated with curcumin. In the isolated lung tissues, a notable increase in oxidative damage was observed, suggesting that curcumin activated cancer pathways via accumulation of ROS. The observed data led the authors to the conclusion that this effect is organ-specific, and the use of curcumin by smokers and ex-smokers may result in the promotion of lung cancer. These findings, once again, show the complexity and uniqueness of the lung carcinogenesis processes.

### 3.2. Drug Repurposing in the Area of Lung Chemoprevention

Many of the cancer-associated targets and pathways play key roles in the pathogenesis of other disorders, successfully treated by existing therapies. Thus, repurposing these therapies for preventing or reverting carcinogenesis processes is one of the most evaluated strategies in the area of chemoprevention.

#### 3.2.1. Anti-Inflammatory Compounds as Chemopreventive Agents

Due to the role of inflammation in general carcinogenesis processes [127] and in tobacco-induced lung cancer initiation and progression [128], the hypothesis of the chemo-preventive effect of NSAIDs became very popular. In fact, anti-cancerogenic effect of aspirin and other NSAIDs has been observed in a variety of in vitro, in vivo cancer models, and confirmed in clinical trials [85]. For aspirin, the most pronounced preventive effect was observed for gastrointestinal cancers, and for stomach cancers [85]. There are some epidemiological data showing NSAIDs’ impact on lung cancer incidence [129]. However, these observations did not have a clear transition to clinical studies, where inconsistent results were observed [130,131,132] For example, the Women’s Health Study (1993–2012) recruited healthy female health professionals (39,876 women, >45 years of age) without previous history of cancer to monitor the effect of the low-dose aspirin (100 mg every other day) use on the cardiovascular health and cancer incidents. Long-term data have shown no overall effect of aspirin on lung cancer incidence [133]. Similarly, the Vital Study showed no effect of pre-diagnostic use of aspirin on the survival rate in patients with lung cancer [130]. Moreover, in this study, the high use of ibuprofen, non-aspirin NSAID, was associated with the 64% increase in lung cancer mortality.

The role of the COX2/prostaglandin E2 (PGE-2) in lung carcinogenesis has been well described. Overexpression of COX-2 is linked with poor outcomes in NSCLC, whereas inhibition of this pathway is shown to reduce lung cancer in animal models [132]. Celecoxib, COX-2 inhibitor, was shown to suppress inflammation process by blocking cigarette smoke condensate-induced expression of NF-kB, COX-2, cyclin D1 and matrix metalloproteinase-9 [134]. Such data provide the rationale for clinical studies with selective COX-2 inhibitors as lung cancer prevention agents. One of such studies [131] has monitored the effect of low (200 mg daily) and high (400 mg twice a day) doses of celecoxib in the pool of smokers and former smokers using Ki-67 as a biomarker. The results showed a statistically significant reduction in Ki-67 expression in smokers (by 1.10%) and in former smokers (by 3.85%). In another study, anticarcinogenic properties of celecoxib (400 mg twice a day) were further confirmed in former-smokers (age ≥ 45, smoking cessation ≥ 1 year), also using Ki-67 expression as a biomarker [82]. In this trial, a more significant reduction in Ki-67 expression (34%) was observed, reaching the levels previously reported in a pilot study in active smokers [135]. In addition, the authors linked the responsiveness of patients to celecoxib treatment with the levels of PGE2, which turned out to be by 2.9-fold higher in responders than non-responders. Finally, they noted the possible shift of arachidonic acid metabolic pathways towards the 15-lipoxygenase-induced conversion to 15(S)-hydroxy-eicosatetraenoic acid, suggesting that this shift can be a compensation mechanism for COX2 inhibition.

Inhibition of COX-2 pathway leads to a subsequent reduction in the levels of the downstream metabolites of arachidonic acid, including prostacyclin (prostaglandin, PGI_2_) that was linked with the reduced rate of metastases [136]. In chemical- or tobacco-induced lung cancer models, supplementation with the prostacyclin analog iloprost lead to a lower rate of lung tumor formation, suggesting utilization of this pathway as a chemopreventive target [136]. Following the success of iloprost in the murine model, a phase II to clinical trial [137] was conducted to investigate its effect in current and former smokers (n = 152) with the sputum cytologic atypia (high-risk population). Iloprost was delivered orally (50 µg twice daily) for six months, and bronchoscopy was performed to monitor any changes in patients. The results show significant improvement in biopsy data for former smokers (5-fold reduction), while no histologic improvement was noted in current smokers [137].

The use of steroid anti-inflammatory agents has also been evaluated for their chemopreventive potential. Particularly, budesonide, a corticosteroid that can be selectively retained by the airway epithelium and accumulates there by anchoring to the long-chain fatty acids [138], inhibited the progression of toxin-induced lung cancer in vivo [139,140,141]. When tested in clinical trials, inhaled budesonide showed no effect on progression rates of bronchial dysplasia [142] and lung nodule sizes [143] in the population of heavy and former smokers. However, in two clinical studies, a more specific group of patients with chronic obstructive pulmonary disease (COPD) presented statistically significant dose-dependent decreased risk of lung cancer associated with inhaled corticosteroids [144,145].

Chang et al. [146] have shown that agonists (thiazolidinediones and 15d-PGJ_2_) of the steroid peroxisome proliferator-activated receptor gamma (PPARγ) promote differentiation of the NSCLC cells, and at higher concentration cause arrest of cell growth and induction of apoptosis. PPARγ is a key regulator of adipogenic differentiation, and its ligands, thiazolidinediones, are used as anti-diabetic agents. In lung adenocarcinoma, the accumulation of somatic mutations can result in inactivation of PPARγ, allowing cancer cells to bypass action of cell cycle control genes, such as p21 [147]. Hence, restoring the function of PPARγ as a cell cycle terminator and promoter of cell differentiation is viewed as a potential anticancer/chemopreventive strategy [148]. For testing this hypothesis, a retrospective study of male veterans with diabetic conditions was conducted, where the effect of thiazolidinedione use on incidence of lung, prostate, and colorectal cancer was assessed [149]. Out of three cancers that were monitored, only results for lung cancer were statistically significant, reaching 33% in rate reduction. Further, oral doses of pioglitazone, an FDA-approved antidiabetic agent of the thiazolidinedione group, delayed the progression to invasive cancer and inhibited tumor load by ≥50% in mouse lung adenocarcinoma model [150]. Similarly, pre-treatment of animals with pioglitazone prevented lung tumor development in the NKK-induced mouse lung cancer model [151]. In another meta-analytic study [152], lung cancer incidence was analyzed in diabetic patients treated with thiazolidinedione (TZD) agents or metformin. In both cases, the risk of developing lung cancer was reduced. However, an increased incidence of metastatic disease and low survival rates were noted for patients who developed lung cancer while on metformin. The protective effect of pioglitazone was also confirmed in Taiwanese study on diabetic patients with COPD/tobacco abuse [153]. For the rest of patients in that study, the protective effect was seen only after a cumulative dose of 15,300 mg. At the same time, a meta-analysis study performed by Bosetti et al. reported a null association between the use of TZDs and lung cancer, while a 20% increase in the risk of bladder cancer was noted [154]. Overall, analysis of these studies suggests that chemoprotective effect of pioglitazone is highly individualized, with the more pronounced benefits in the subpopulation of patients with the increased risk of lung cancer, such as COPD patients and heavy smokers. This hypothesis was tested by the latest clinical study in subjects at increased risk for lung cancer. However, use of pioglitazone for 6 months did not provide any benefits in participants, with no significant differences observed in the treatment group vs placebo group and in current smokers vs former smokers [155].

#### 3.2.2. Modulators of Signaling Pathways

One of the general chemopreventive strategies includes inhibition of the PI3K/Akt/mTOR pathway that is responsible for the proliferation of cancer cells. Gustafson et al. showed that PI3K is activated in a cytologically normal proximal airway of smokers [156] and its activation can be used as a monitoring tool in a high-risk population. They also noted a simultaneous activation of protein kinase C (PKC), a downstream component of PI3K, whereas no loss of phosphatase and tensin homolog (PTEN) or increase in PI3K copies was observed. These findings, specific for lung tumorigenesis, provide data for needed chemopreventive strategies. As described elsewhere [157,158], inhibitors of PI3K/Akt/mTOR are being extensively investigated for the ability to revert/prevent cancer development in preclinical and clinical settings [159,160,161]. Although some preclinical data suggested the potential benefit of mTOR inhibitors [162,163], significant side effects associated with their use prevented the development of clinical trials with this class of drugs. Metformin, another inhibitor of mTOR [157], has shown some potential for chemoprevention in in vivo models [164], further supported by some meta-analytical and epidemiologic studies in type-2 diabetic patients [165,166,167,168,169,170,171]. However, selected studies report no effect of metformin on the development of several types of cancers, including lung cancer, in diabetic patients [172]. As mentioned earlier, the development of lung cancer while on metformin was associated with more aggressive disease and poor prognosis [152]. Myo-inositol, an inhibitor of the PI3K pathway, also showed a chemoprotective effect in vitro [173,174], and in carcinogen-induced murine lung cancer models [175,176]. The pilot human trial (26 subjects) revealed a statistically significant regression of dysplasia in participants treated with myo-inositol [177]. Mechanistic and in vivo studies suggested the benefit of using of myo-inositol in Kras mutated high-risk smokers [178,179].

**Figure 4:**
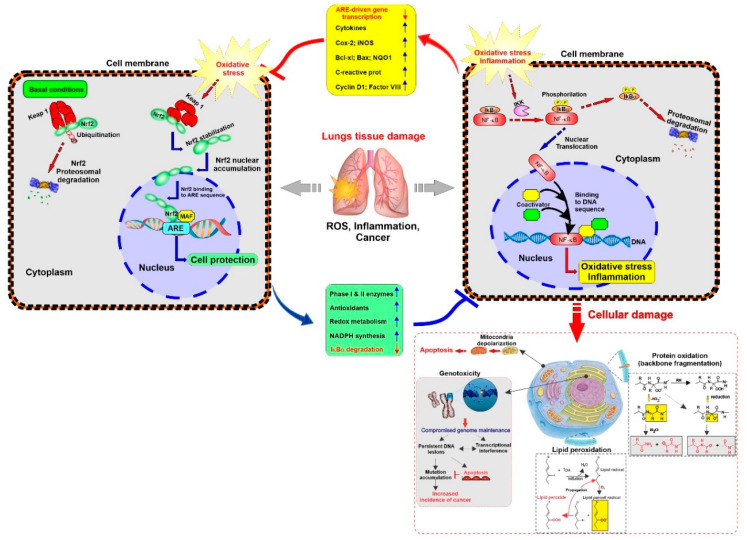
Reactive oxygen species (ROS) promote cellular inflammatory response and oxidative damage, which may impact cell and tissue viability in the lungs. The figure is a schematic illustration depicting the primary point of mutual interference between Nrf2 and NF-κB. Note the cellular regulation of Nrf2 and NF-κB under normal and stressed conditions. Both Nrf2 and NF-κB are nuclear transcription factors that balance and counteract each other’s activity. Note also that the predominant activation of the NF-κB pathway through ROS exposure (generated either endogenously and/or exogenously) promotes genotoxicity, lipid peroxidation, and protein degradation leading to cellular and tissue damage.

The Nrf2/Keap1 signaling pathway is one of the key cell defense mechanisms against oxidative and xenobiotic stress (Figure 4). As was shown earlier, activation of the Nrf2 pathway may be an effective chemoprevention strategy, as it promotes restoration of the redox balance and significantly reduces stress induced by the presence of the environmental toxins, including tobacco smoke [180]. At the same time, cancer cells, especially chemo resistant ones, are characterized by the dysregulation of Nrf2-associated pathways [60]. Thus, careful timing of the Nrf2-based treatment is required to achieve a chemo-protective effect. Many natural products are shown to modulate Nrf2. However, their use is often associated with off-target activity that can be incompatible with the long-term use of chemo-preventive agents. Hence, synthetic modulators of this pathway may have more potential as clinical agents. The design and evaluation of Nrf2 modulators are comprehensively reviewed by Sova et al. [181], where the authors provide full information on the current status of this area of chemo-preventive agents.

Statins, drugs used for lowering low-density lipoprotein cholesterol levels, were shown to suppress tumor cell growth in several in vitro and in vivo models. The proposed mechanisms of the observed effect are based on their ability to trigger tumor-specific apoptosis and inhibit the proteasome pathway [182,183]. In clinical settings, statins were shown to protect patients with COPD against the development of lung cancer, possibly by reducing the inflammation-induced stress on the organ through modulation of the NF-kB/STAT3 pathway. In this study [184], out of 42,802 patients with the aforementioned chronic conditions, 10,086 (30%) were using different statin-based agents. The control group patients used non-statin agents, as well as metformin and aspirin. As an outcome, the authors reported 63% reduction in lung cancer incidence in COPD patients who used rosuvastatin, atorvastatin, and simvastatin. The observed effect of statins was dose-dependent. Similarly, a 55% reduction in the risk associated with lung cancer development was observed in the retrospective case-control study of US veterans (483,733 patients). This effect was observed after six months of use of statins, was seen across different age and racial groups, and was irrespective of the presence of diabetes, smoking, or alcohol use [185]. However, a meta-analysis of 21 observational studies and 8 randomized controlled trials performed by Deng et al. [186] showed no connection between the use of statin and lung cancer rate in the general population and in the elderly people. This outcome can be explained by the lack of a classification of the statin therapy used in the analyzed studies. This limitation seems significant since only selected statins were shown to have a chemo-preventive potential, while others, like lovastatin and fluvastatin, had no association with the reduction in the lung cancer risks [184].

Recently, a few reports have published data on the link between the use of the angiotensin receptor blockers (ARBs) and angiotensin-converting-enzyme (ACE) inhibitors and the risk of lung cancer. The chemo-preventive potential of ARBs was first reported in the meta-analysis study by Zhang et al. [187], where they analyzed 5 retrospective studies (total of 3,283,285 patients) and 3 case-control studies (total of 1,047,769 patients). The results show a ~20% reduction in the incidence of this type of cancer associated with the use of ARBs. At the same time, the large cohort studies in United Kingdom and Denmark found no effect of ARB use on lung cancer risk [188,189]. Finally, the use of ACE inhibitors has been linked with the statistically significant increase in lung cancer incidences, especially in a subgroup of patients with over ten years of drug use [188,190].

## 4. Examples of New Chemopreventive Strategies

### 4.1. Alteration of Paracrine Signaling of Endothelial Cells as a Tertiary Cancer Prevention Strategy

Endothelial cells are part of every tissue in a human body, and signaling induced by these cells is an essential part of every organ’s microenvironment. Under normal conditions, quiescent endothelial cells play protective role preventing disbalance of pro-inflammatory signaling and inhibit tumor growth and metastasis, as was confirmed in vitro and in vivo [191]. Chronic exposure of lungs to tobacco smoke, as well as other environmental toxins, promotes accumulation of genetic and epigenetic changes, some leading to the formation of the dysfunctional endothelial cells (DEC) [192]. These cells are characterized by aberrant integrin and extracellular expression, abnormal responses to oxidative stress, and the mechanical microenvironment. Dysfunctional endothelial cells promote activation of NF-kB pro-inflammatory signaling, stimulating cancer cell apoptosis while pre-selecting for more aggressive cancer cells. In addition, the DEC environment results in a 33% increase in the invasiveness of the A549 lung cancer cells. In the in vivo settings, the presence of dysfunctional endothelial cells causes a reduction in the size of primary Lewis lung carcinoma tumors but initiated spontaneous metastasis [192]. These findings suggest that the use of chemopreventive agents targeting restoration of the NF-kB controlled signaling in endothelial cells may prevent carcinogenic and metastatic processes in patients with a history of lung cancer.

### 4.2. Synthetic Lethality Strategy

The concept of synthetic lethality is based on the hypothesis that by inhibiting a cancer-specific interaction between two or more genes and pathways involved in the survival of cancer cells, one can achieve highly selective cancer cell death [69]. In their paper, Huang et al. use interconnection between Ras, TRAIL, and Smac. Constitutive expression of Ras signaling is characteristic of many cancers. In NSCLC, it is often associated with the KRAS mutations that are present in up to 40% of atypical adenomatous hyperplasia lesions. TRAIL, a TNF-related apoptosis-inducing ligand, was shown to induce apoptosis selectively in cancer cells, with little or no toxicity in vivo (cynomolgus monkeys and chimpanzees) [193,194] or in Phase I clinical trials [195]. A combination of the TRAIL and Smac mimic (allows overcoming KRAS-induced survival in cancer cells) was shown to induce strong apoptosis in premalignant lung adenoma cells and inhibit the formation of KRAS-induced lung cancer in vivo [76]. These outstanding results are further signified by the fact that KRAS mutations are linked with resistant lung cancer and poor prognosis in patients.

### 4.3. Modulation of Lung Microbiome

Many reports show a significant shift of lung microbiome composition in patients with lung cancer compared to patients with no history of lung cancer. In particular, sputum samples of patients with lung cancer were enriched with *Streptococcus viridans*, *Ganulicatella adiacens*, *Escherichia coli*, *Enterococcus sp 130*, and *Acinetobacter juni* [196]. The bacterial composition of these samples was also linked with the stage of the disease, suggesting the potential utilization of bacterial biomarkers for identification of the status and stage of lung cancer [197]. Furthermore, in vivo experiments identified a strong correlation between the use of ampicillin, vancomycin, neomycin sulfate, and metronidazole in mice with Lewis lung cancer tumors and shortened median survival of animals. In humans, the abnormal composition of microflora, in some cases induced by the use of antibiotics, results in resistance to checkpoint inhibition therapy [198]. Prolonged exposure to tobacco smoke leads to chronic inflammation in the lungs that results in unstable microbial community [199], with the reduced within-sample taxonomic diversity (alpha diversity). Hence, one of the chemo-preventive strategies can focus on maintaining the biodiversity of the lung microbiome via implementation of a special diet and the use of vitamins and probiotics. Possibly, the observed chemo-preventive effect of a diet enriched with vitamins A and E, as discussed above, can be ascribed to the modulation of the microbiome’s health. The metabolic effect of dysbiosis and genotoxicity of several bacterial molecules is known to play a certain role in the initiation of carcinogenesis [200]. Hence, another chemo-preventive strategy can utilize the use of the ligands capable of inhibiting the biosynthesis of harmful molecules. Such ligands should have minimal bactericidal/bacteriostatic effect to avoid further disbalance of species composition, similarly to the use of quorum sensing inhibitors that reduce the production of bacterial virulence factors without affecting the well-being of microorganisms [201].

## 5. Conclusions

Lung cancer remains to be one of the deadliest cancer types, mostly due to the asymptomatic early stages of the disease. Tobacco-induced changes in the lung tissue accumulate over a prolonged period of time before proceeding to the cancerous stage. Hence, effective chemo-preventive strategies can significantly reduce the incidence of lung cancer and mortality associated with this condition. At the moment, a plethora of in vitro, in vivo, and even epidemiological evidence did not translate to robust clinical outcomes, highlighting the heterogenicity of the conditions associated with the initiation and progression of lung tumors. At the same time, specific chemo-preventive strategies, such as the use of statins in COPD patients and anti-inflammatory agents in selected subgroups of patients, were very successful, suggesting a need to develop individualized approaches for prevention of lung cancer development (Table 2). In addition, we believe that clinical studies in this field need to be designed for a specific subpopulation of participants, since variation in the responses can vary significantly between current smokers, former smokers and non-smokers. Further, the synthetic lethality approach, if confirmed in the clinical setting, will offer highly effective non-toxic treatment of high-risk patients with a genetic predisposition to lung cancer.

## Figures and Tables

**Table 1:** Role of selected phytochemicals in chemoprevention.

Affected Pathways	Proposed Mechanism of Action	Phytochemicals	Models
Anti-oxidant	Inhibition of PKA-induced signaling and PKA-induced generation of ROS Suppression of ROS production and modulation of insulin-like growth factor -I/pathway	Resveratrol (grape) Caffeic acid Curcumin Plumbagin Honokiol Lycopene (tomato)	Multiple cancer lines [103] in vitro Smoke-induced carcinogenesis model in ferrets [117]
Modulation of invasion and migration of cancer cells	Inhibition of MMP-2	Plumbagin	Human A549 lung cancer cells [104]
Modulation of the immune system	Potentiation of NK cell lysis via NKG2D pathway	Resveratrol	Clinical trial [118]
Induction of apoptosis	Reduction of the mitochondrial membrane potential and release of cytochrome c Induction of apoptosisi and cell cycle arrest at the G1 phase Downregulation of Bcl-2 and NF-κB pathway Modulation of PI3K/AKT/mTOR signaling pathway	Polyphyllin D (Chinese medical herb *Paris polyphylla*) Ganoderic acid T (Chinese medicine herb Ganoderma lucidium) Green tea polyphenols (EGCG) Green tea polyphenols (EGCG) Polyphyllin VII (Chinese medical herb *Paris polyphylla*)	Lewis lung cancer cells [119] in vitro NSCLC (A549, NCI-H460) [120] cells in vitro Tobacco carcinogen-induced (NKK-induced) lung tumors in A/J mice [121] A549 human lung cancer in vitro [122]
Alteration of epigenetic alterations in cells	Modulation of DNA methylation and chromatin modeling	Green tea polyphenols	H460 lung cancer cell line [123] A549 and H460 human lung cancer cell lines in vitro and lung cancer xenograft of A549 in BALB/c nude mice models in vivo [124]
Inflammation	Modulation of the IL-10 and TGF-β	Water extract of ginseng and astragalus	A549 cells in vitro and LLC-allografted mice model [125]
Glucose Metabolism	Inhibition of isoform 5 of lactate dehydrogenase	Crocetin (saffron)	human A549 lung cancer cells [126] in vitro

Abbreviations: protein kinase A, PKA; reactive oxygen species, ROS; insulin-like growth factor, IGF; matrix metallopeptidase 2, MMP2; natural killer cells, NK cells; B-cell lymphoma 2, BCL-2; nuclear factor kappa-light-chain-enhancer of activated B cells, NF-κB; the phosphoinositide 3-kinase, PI3K; protein kinase B, AKT; mammalian target of rapamycin, mTOR; deoxyribonucleic acid, DNA; interleukin 10, IL-10; transforming growth factor beta, TGF-β; epigallocatechin gallate, EGCG; non-small cell lung cancer, NSCLC; National Cancer Institute, NCI; nitrosamine-4-(methylnitrosamino)-1-(3-pyridyl)-1-butanone, NKK; Lewis lung carcinoma, LLC.

**Table 2:** List of selected lung cancer chemoprevention trials listed in the review.

Class of Chemopreventive Agents	Chemopreventive Agent	Trial	Suboplulation of Patients	Reported Outcomes
Vitamins and minerals	⍺-tocopherol, β-carotene	ATBC Study (Finland, 1994)	29, 133 male smokers (≥ 5 cigarettes/day)	17% increase in the lung cancer rate and 8% increase in the overall death rate [91]. The follow-up study showed reduction in the risk of those who quitted smoking [92].
	β-carotene, retinol	CARET Study (USA, 1985)	18,344 current and ex-smokers, male and female; workers with history of asbestos exposure	Study was stopped 21 months earlier due to the higher incidence of lung cancer, higher rate of cardiovascular-related, mortality and overall mortality [93].
	β-carotene	Physician’s Health Study (USA, 1982)	22,071 male physicians, current smokers (11%) or former smokers (39%)	After 12 years, no effect was observed on any of the outcomes (malignant neoplasia, cardiovascular diseases, and death from all causes) [95]
	Selinium	The Nutritional Prevention of Cancer Trial	1312 participants	Lung cancer was the secondary end point, with the pronounced benefit observed only in the subgroup with the low baseline of Se in serum [101].
	Selinium	Secondary lung tumor prevention	1772 patients with resected stage I non-small cell lung cancer	No chemoprevention benefit was observed [102]
Phytochemicals	Green tea or polyphenon E	NCT00363805 (USA, 2004)	195 patients, current or ex-smokers with Chronic Obstructive Pulmonary Disease	No results were published
	Green tea, black tea	NCT02719860 High Tea Consumption on Smoking Related Oxidative Stress (USA, 2004)	154 participants, current and ex-smokers	No results were published
	Glucobrassicin-rich Brussels sprouts	NCT0299939 Glucobrassicin-Brussel Sprout Effect on D10 Phe Metabolism (USA, 2016)	48 participants, current and ex-smokers	Ongoing study
	Sulforaphane	NCT03232138 Clinical Trial of Lung Cancer Chemoprevention With Sulforaphane in Former Smokers	72 participants, current and ex-smokers	Ongoing study
Anti-innflammatory agents	Low-does aspirin (100 mg daily)	Women’s Health Study (USA, 1993)	39,876 healthy female health professionals	No effect on lung cancer incidents [133]
	NSAID’s and vitamin B	The VITamins And Lifestyle (Vital) Study	77,738 men and women	No effect of pre-diagnostic use of aspirin on the survival rate in patients with lung cancer [202]. High use of ibuprofen was associated with the 64% increase in lung cancer mortality.
	Low (200 mg daily) and high (400 mg twice a day) doses of celecoxib	Biological activity of celecoxib in the bronchial epithelium of current and former smokers (USA, 2010)	204 patients, smokers and former smokers	High-dose showed statistically significant reduction in Ki-67 expression in smokers (by 1.10%) and in former smokers (by 3.85%) [131]
	Celecoxib (400 mg daily)	Lung cancer chemoprevention with celecoxib in former smokers (USA, 2010)	137 participants, former smokers (≥ 30-pack-years of smoking; ≥ of sustained abstinence of smoking)	Significant reduction in Ki-67 expression (up to 34%) in the celecoxib-arm. Beneficial results were linked with the higher levels of PGE2 in responders [82].
	Iloprost	Effect of oral iloprost on endobronchial dysplasia in former smokers(USA, 2012)	152 subjects with the sputum cytologic atypia (current and former smokers)	Significant improvement in biopsy data for former smokers, no effect was observed for current smokers [137].
	Budenoside(1600μg daily for 6 months)	A randomized phase IIb trial of pulmicort turbuhaler (budesonide) in people with dysplasia of the bronchial epithelium.	112 smokers with one or more sites of bronchial dysplasia	In smokers, inhaled budenoside showed no effect on regression of bronchial dysplastic lesions or prevention of new lesions [142].
	Budenoside(800μg twice daily for 12 months)	Randomized phase II trial of inhaled budesonide versus placebo in high-Risk individuals with CT screen–detected lung nodules (USA, 2011)	202 subjects, current and former smokers with CT-detected lung nodules that were persistent for at least 1 year	No effect was detected on lung nodules sizes, although per-lesion analysis showed effect on a regression of existing target nodules. No effect was observed for peripheral nodule sizes [143].
	Triamcinolone, beclomethasone, flunisolide, fluticasone	Inhaled corticosteroids and risk of lung cancer among patients with chronic obstructive pulmonary disease. (USA, 2001)	10,474 subjects with a diagnosis of COPD and no history of lung cancer	A dose-response decrease in risk of lung cancer was observed [144].
	Fluticasone and budenoside	Effect of inhaled corticosteroids against lung cancer in female patients with COPD: a nationwide population-based cohort study (Taiwan, 2009)	13,868 female COPD patients	A reduction of 1.5 fold in lung cancer incidence rate was observed in patients treated with the inhaled corticosteroids [145].
PPARγ agonists	Pioglitazone	A Randomized Phase II Trial of Pioglitazone for Lung Cancer Chemoprevention in High-Risk Current and Former Smokers (USA, 2019)	92 subjects with the sputum cytologic atypia (current and former smokers)	Slight improvement in worst biopsy scores, dysplasia index, and average score was observed in former smokers. No protective effect was observed in current smokers [155].
Modulators of mTOR pathways	Myo-inositol (18 g daily for 3 months)	A phase I study of myo-inositol for lung cancer chemoprevention (Canada, 2006)	26 participants, smokers (≥ 30-pack-years of smoking) with one or more sites of bronchial dysplasia. Dose -escalation study had 16 participants, chemoprevention study was done in a group of 10 patients	Significant regression of dysplastic lesions was observed [177].
Statins	Lovastatin, fluvastatin, rosuvastatin, simvastatin, atorvastatin, and pravastatin	A population-based cohort study to evaluate effect of stating against lung cancer in COPD patients (Taiwan, 2012)	43,802 COPD patients	Statins, except lovastatin and fluvastatin, produce dose-dependent chemopreventive effect in studies COPD group of patients [184].
	Statins	A retrospective case-control study of US veterans evaluation the effect of statins on the risk of lung cancer in humans (USA, 2004)	483,733 subjects	Statins showed protective effect against the development of lung cancer [185].
Antihypertensive agents	Angiotensin receptor blockers, diuretics beta-blockers, angiotensin-converting enzyme inhibitors and calcium channel blockers.	A retrospective cohort study to analyze an association between the use of angiotensin receptor blockers and cancer (UK, 2010)	1,165,781 patients that were prescribed antihypertensive agents	Angiotensin receptor blockers, diuretics beta-blockers had no effect on the rate of lung cancer incidences. However, angiotensin-converting enzyme inhibitors and calcium channel blockers were associated with the higher incidences of lung cancer [189].
	Angiotensin receptor blockers	A retrospective nationwide cohort study: use of angiotensin receptor blockers and the risk of cancer (Denmark, 2006)	107,466 ARB users (≥ 35 years)	No effect of Angiotensin receptor blockers on lung cancer incidences [188].
	Angiotensin-converting enzyme inhibitors, angiotensin receptor blockers	Population based cohort study to analyze an association between the use of angiotensin-converting enzyme inhibitors and cancer (UK, 2016)	992,061 patients newly treated with any hypertensive agents.	Use of angiotensin-converting enzyme inhibitors was associated with the higher incidences of lung cancer [190].

## Abbreviations

4-ABP	4-aminobiphenyl
8-oxoG	8-oxi-7,8-dihydroguanine
AAH	Atypical adenomatous hyperplasia
ACE	Angiotensin-converting-enzyme
Ach	acetylcholine
ADC	Adenocarcinoma
ALK	Anaplastic lymphoma kinase
ARBs	Angiotensin receptor blockers
ARE	Antioxidant response element
ATBC	Alpha-Tocopherol Beta Carotene
B(a)P	Benzo[a]pyrene (B[a]P)
CARET	β-Carotene and Retinol efficacy Study
CDC	Center for Disease Control and Prevention
CIS	Squamous dysplasia and carcinoma
COPD	Chronic obstructive pulmonary disease
COX-2	Cyclooxygenase-2
DEC	Dysfunctional endothelial cells
EGFR	Epidermal growth factor receptor
EVALI	E-cigarette, or vaping, product use–associated lung injury
HNE	Trans-4-hydroxy-2-nonenal
IGF	Insulin-like growth factor
IEN	Intraepithelial neoplasia
Keap-1	Kelch-like ECH-associated protein 1
MDA	Malondialdehyde
nAChR	nicotinic acetylcholine receptor
NF-κB	Nuclear factor-κB
NNAL	Nnitrosamine alcohol
NNK	Nitrosamine ketone
NNN	N-nitrosonicotine
Nrf2	Nuclear factor E2-related factor 2
NSAIDs	Non-steroidal anti-inflammatory drugs
NSCLC	Non-small cell lung cancer
p38 MAPK	p38 mitogen-activated protein kinases
PGE-2	COX2/prostaglandin E2
PPARγ	Peroxisome proliferator-activated receptor gamma
ROS	Reactive oxygen species
SCLC	Small cell lung cancer
SCC	Squamous cell carcinomas
Se	Selenium
TKIs	TK inhibitors
TZD	Thiazolidinedione
